# A dynamic social norm messaging intervention to reduce meat consumption: A randomized cross-over trial in retail store restaurants

**DOI:** 10.1016/j.appet.2021.105824

**Published:** 2022-02-01

**Authors:** Elif Naz Çoker, Rachel Pechey, Kerstin Frie, Susan A. Jebb, Cristina Stewart, Suzanne Higgs, Brian Cook

**Affiliations:** a Nuffield Department of Primary Care Health Sciences, University of Oxford, Radcliffe Primary Care Building, Radcliffe Observatory Quarter, Woodstock Road, Oxford, OX2 6GG, UK; b The School of Psychology, University of Birmingham, Edgbaston, Birmingham, B15 2TT, UK

**Keywords:** Meat consumption, Social norms, Dynamic norms, Intervention, Social influences, Dietary choice

## Abstract

Perceptions of social norms around eating behavior can influence food choices. Communicating information about how others are changing their eating behavior over time (dynamic descriptive social norms) may motivate individuals to change their own food selection and consumption. Following a four-week baseline period, 22 in-store restaurants of a major retail chain across the UK were randomized to display a dynamic descriptive social norm message intended to motivate a shift from meat-to plant-based meals either during the first two, or last two weeks of the four-week study period. A linear regression model showed there was no evidence of an effect of the intervention (β = -0.022, p = .978, 95% CIs: −1.63, 1.58) on the percentage sales of meat- vs plant-based dishes. Fidelity checks indicated that adherence to the intervention procedure was often low, with inconsistencies in the placement and display of the intervention message. In four stores with high fidelity the estimated impact of the intervention was not materially different. The lack of apparent effectiveness of the intervention may reflect poor efficacy of the intervention or limitations in its implementation in a complex food purchasing environment. The challenges highlighted by this study should be considered in future design and evaluation of field trials in real-world settings.

## 1 Introduction

Meat consumption in the UK has increased from 69 kg to 79 kg per year per capita in the past fifty years, while meat production has almost doubled in the same timeframe from 2.2 to 4.1 million tons per year (Ritchie & Roser, 2017). However, it seems that this rise in meat consumption peaked in the mid-2000s, with average daily meat consumption per capita decreasing by 17% in a recent decade (Stewart, Piernas, Cook, & Jebb, 2021). Despite this reduction, current levels of consumption are still well above the recommended amounts. The EAT-Lancet Commission on Food, Planet and Health report states that a healthy and sustainable diet should have no more than 26 kg of meat including red meat, poultry and fish per year (Willett et al., 2019). The UK National Food Strategy recommends that national meat consumptionshould decrease by a further 30% over the next ten years (Dimbleby, 2021).

High intake of red and processed meat has been found to be associated with a variety of health risks (Papier et al., 2021; Rouhani, Salehi-Abargouei, Surkan, & Azadbakht, 2014), while livestock farming is responsible for about 14.5% of all greenhouse gas emissions (Gerber et al., 2013; Godfray et al., 2018; Lazarus, McDermid, & Jacquet, 2021) and uses more land and freshwater compared to plant-based food production (Ranganathan et al., 2016). Given the evidence for the negative health and environmental impacts of high levels of meat consumption, how can individuals be persuaded to significantly reduce their intake? Recent research indicates that meat consumption is one of the domains where individuals often do not make decisions in isolation and are open to being influenced by how others behave in social settings (Cheah, Shimul, Liang, & Phau, 2020). Appealing to individuals’ perceptions of social norms around meat consumption, therefore, presents an opportunity to motivate a shift away from animal-based to plant-based foods.

Social norms can be described as a set of informal, unwritten rules or standards for behavior that can constitute effective decision heuristics for finding a contextually appropriate way to behave, especially in new and unfamiliar situations (Brachem, Krüdewagen, & Hagmayer, 2019; Cialdini & Goldstein, 2004). Descriptive social norms are the generally accepted ways of behaving that are commonly observed and can constitute a pattern for an individual to inform their own behavior (Brachem et al., 2019; Cialdini & Goldstein, 2004), while injunctive norms indicate ideal, desired or approved ways of thinking and acting that may not match the observed behavior of the social group.

A systematic review examining the communication of information about descriptive eating norms has found that the majority of studies reported changes in the eating behaviors of participants exposed to the norms (Robinson, Thomas, Aveyard, & Higgs, 2014). However, the evidence relating to the use of social norm messages to increase the consumption of plant-based foods and reduce meat consumption is sparse. The majority of previous studies on social norms and plant-based eating have used static norm messaging and focused on increasing fruit and vegetable intake rather than encouraging a switch from meat-to plant-based options (Collins et al., 2019; Gonçalves, Coelho, Martinez, & Monteiro, 2021; Huitink, Poelman, van den Eynde, Seidell, & Dijkstra, 2020; Mollen, Rimal, Ruiter, & Kok, 2013; Thomas et al., 2017). Two systematic reviews about sustainable eating behaviors (Taufik, Verain, Bouwman, & Reinders, 2019) and reducing meat consumption (Harguess, Crespo, & Hong, 2020) found only one study that used social norm messaging as an intervention to encourage a switch from meat-to plant-based meals (Sparkman & Walton, 2019). This study, followed by further experiments by the same group of researchers (Sparkman, Weitz, Robinson, Malhotra, & Walton, 2020), used dynamic descriptive norms – i.e. social norms that describe how people's behaviors are changing over time – to communicate a recent increase in the proportion of people reducing their meat consumption. The studies included online experiments as well as field experiments in a university campus café and a restaurant and observed both an increase in intentions to reduce meat consumption and an actual percentage increase in the purchase of plant-based meals. The findings of the study highlighted the importance of location, visibility, and target population of dynamic norm messages in their effectiveness. The use of dynamic norms that highlight a shift in existing behavior seemed to provide a unique opportunity in encouraging increased intake of plant-based dishes in cultures where meat consumption is the existing norm. Communicating that an increasing number of individuals are opting for plant-based dishes, made it more likely for individuals to think that this is an ongoing trend that could replace the existing norm, and they were thus more inclined to change their behavior, “pre-conforming” to the future norm.

Most studies that looked at the effects of social norms on eating behavior have recruited adolescents or university students with little evidence available for groups that are more representative of the general population (Collins et al., 2019; Mollen et al., 2013; Robinson, Fleming, & Higgs, 2014; Robinson, Harris, Thomas, Aveyard, & Higgs, 2013; Stok, de Vet, de Ridder, & de Wit, 2016), while few looked at food purchasing behavior beyond intentions or self-reports in real-life settings (Garcia et al., 2021; Thomas et al., 2017).

The present study tested a dynamic, descriptive social norm intervention in a retail chain's in-store restaurants with the primary objective of reducing purchases of meat-based meals and increasing the sale of plant-based meals, offering the opportunity to observe the effect on actual purchasing patterns of restaurant-goers across the UK.

## 2 Methods

### 2.1 Setting

The intervention was conducted in 22 in-store restaurants of a retail store chain that specializes in the sale of non-food related products in the UK (located across England, Wales, Scotland and Northern Ireland). The stores are all located in the immediate periphery of urban centers. According to the data communicated by the retail store chain to the research team, the in-store restaurants attract about one-third of all store-goers, are advertised as family-friendly, and serve millions of meals across the country per year, attracting a broad cross-section of the population. The restaurants have a cafeteria-style setting, where meal options are presented in a buffet visible to customers with servers behind the counter who serve the customers their chosen dishes. Customers are not allowed to serve the main meals themselves, but are able to pick up drinks and snacks. The food items on offer are intended for immediate consumption in the adjacent seating area, and no take-away options are offered. The atmosphere of the restaurants is fast-paced and casual, appealing to a general UK audience, and the food offering does not specialize in local, organic or non-GMO products. The meals are affordably priced, with main meals and included sides costing around £4.50 – £5.50.

### 2.2 Study design

The sample size for the study was pragmatically determined, comprising the maximum number of stores that the retail store chain agreed to make available to us for participation (N=22). We used a cross-over design with stores randomized to the order in which they completed the intervention and control condition. To control for the possibility of some customers visiting multiple stores and being exposed to different study conditions, the stores were sorted into geographic clusters, and stores in the same cluster were allocated to the same order of study conditions (i.e., intervention first or control first).

The study took place June 17th - July 14th, 2019. Following a 4-week baseline period (May 20th - June 16th, 2019), half of the stores were allocated to an intervention-first arm (social norm message displayed for two consecutive weeks, followed by two weeks of no-message control) and the other half to a control-first arm (no-message control for two consecutive weeks, followed by two weeks of social norm message display).

### 2.3 Social norm messaging

The intervention consisted of a dynamic social norm message displayed on the digital menu and information screen boards in the cafeteria and other locations in the store that are normally used to advertise meals. The phrasing of the social norm message was developed by the research team in consultation with the retail partner following focus group discussions. A total of 28 participants (20 females, 8 males) took part in one of four focus groups. Participants were aged between 19 and 47 years (mean = 25.4 years, SD = 6.6), and were undergraduate or postgraduate students (n = 13), or in full- or part-time employment (n = 15). The discussions explored how people's reactions differ in response to static versus dynamic norms, what constitutes a relevant reference group for the target population, and which adjectives best describe a plant-based meal option. Clarity, believability, and ability to influence were taken into consideration**.** The final social norm message (“More and more [retail store name] customers are choosing our veggie options”) appeared in a green circle on the top right corner of the image displayed on the digital boards, covering 1/8th of the screen, and was animated with a small stretching and wobbling effect to draw customers' attention further (NB: We are unable to provide the visuals of the message and the digital boards in this manuscript due to the confidentiality agreement with our retail partner). The message was displayed alongside a picture of the vegetarian breakfast option during the breakfast service hours, and a vegetarian main meal during the remaining trading hours on digital screens that were located at three locations: the store entrance, the restaurant entrance, as well as on one of the digital menu screens directly above the serving counter.

Besides the inclusion (intervention) or exclusion (control) of the social norm message on digital boards, we requested all other aspects in the in-store restaurant setting, such as meals advertised, branding, images, rotation frequency and duration on the digital screens to be unchanged throughout the study period. As all participating restaurants were managed centrally by the retail partner, they showed great consistency in the type of meals served and menu offerings.

### 2.4 Measures

Restaurants reported their sales on a weekly basis, for a total of eight weeks, from till data recording the number of items sold of each meal on offer. For the purposes of the study, only the main meals on the breakfast and lunch menus and their plant-based equivalents advertised alongside the social norm messages were included in the total number of sales. Sides, salads, soups, and desserts were excluded from the analysis. A meal was categorized as meat- or plant-based at the analysis stage based on the menu titles and the source of the central item of the dish. Plant-based meat-alternatives that were similar in taste, look, and texture to their meat counterparts constituted the central item in plant-based options. The compared meals were similar in terms of portion size, presentation and accompanying sides. The prices of the meat- and plant-based main meals were also very similar, with a 10-20p lower price for plant-based versions. The first four weeks of reported sales constituted the baseline and were averaged to reflect the mean sales of meat- and plant-based dishes during this period, and the same was done for the two-week intervention and control periods.

### 2.5 Intervention fidelity

Nine out of 22 stores were visited once during the intervention period and once during the control period by the research team or trained community researchers for fidelity checks. The restaurants were selected so that each of the geographic clusters were represented. Equal numbers of visits were intended to be made to restaurants allocated to the intervention-first and control-first conditions, but a miscommunication with one of the data collectors resulted in fidelity checks being performed on six intervention-first and three control-first locations. The checks were conducted during mid-morning hours to be able to observe adherence during both breakfast and lunch hours to see the message displayed alongside both the vegetarian breakfast and vegetarian main meal options. Adherence to the intervention procedure was checked on six aspects: whether the three screens located at the store entrance, in-store restaurant entrance, and restaurant menu board displayed the social norm message for both breakfast and lunch time. Restaurants were given a fidelity score out of six, with those scoring 5–6 points classified as having high fidelity, 3–4 points as medium fidelity, and 1–2 points as low fidelity.

### 2.6 Data analysis

The statistical analysis plan specifying hypotheses and primary and secondary outcomes was pre-registered on OSF (https://osf.io/3vrqj/) before any data analysis was conducted. Diverging from the pre-registration, in order to account for the fluctuations in overall sales volume across the weeks, the decision to transform mean sales into percentage values to reflect the proportion of meat- and plant-based sales was taken during data cleaning prior to analysis. The data analysis was conducted using SPSS version 27 (IBM Corp, 2020). The transformed mean percentage values of meat- and plant-based meal sales across three time periods for each restaurant were checked for normality with histograms, Q-Q plots and Shapiro-Wilk tests. After confirming that the data were distributed normally, the data analysis proceeded with parametric tests.

Prior to exploring the effect of the intervention on plant-based meal sales, preliminary analyses looked at whether there were any baseline differences between restaurants, any carry-over effects in intervention-first condition restaurants, and any chronological changes (that could be due to factors such as weather changes, cultural trends, etc.) that might alter customers’ dietary choices.

In order to assess whether the intervention had any carry-over effects into the control period of the intervention-first condition, we compared the differences in the percentage of plant-based meal sales between the intervention period and the control period between the two intervention orders using independent samples t-tests.

Data checks indicated that there was an increase in the overall volume of sales over time in the restaurants, regardless of intervention allocation. To ensure that this did not influence the percentage of plant-based meal sales, the difference between the percentage of plant-based sales in the first and second two-week periods were examined using paired samples t-tests to see if there was a change over time, irrespective of intervention allocation of the restaurants.

A linear regression model was used to predict the effect of the intervention, with restaurants as random effects. The percentage of plant-based meal sales was regressed on condition (intervention versus control), intervention order (intervention first versus control first), and number of plant-based meal options available to purchase (four versus five), all entered as dummy variables. The cross-over design was maintained, and baseline sales were not entered into the model since there were no baseline differences between groups. Time of sale was also not entered into the model since there was no evidence of a period effect.

The effect of the intervention fidelity was examined with an independent samples *t*-test comparing the difference in percentage of plant-based meal sales between intervention and control periods for each restaurant. Since intervention fidelity data was only available for nine out of 22 restaurants, and only four out of these scored medium/high, associated effects were examined in a separate model where percentage of meal sales in only these four restaurants was regressed on condition, intervention order, and number of plant-based meal options available to purchase.

## 3 Results

### 3.1 Baseline characteristics of stores

Independent samples t-tests showed very little variability in the proportion of plant- and meat-based meal sales across stores. There were no significant differences in the mean percentage sales of plant-based meals between the restaurants allocated to the control first (M=.096, SD=.024, 95% CIs: 0.080, 0.112) and intervention first (M=.105, SD=.024, 95% CIs: 0.089, 0.121) arms in the four weeks before the study; (t(20) = -0.90, p = .376, Cohen's d = -.386), suggesting the control and intervention stores were well matched in this respect.

### 3.2 Carry-over effects

There was no evidence of carry-over effects from the intervention into the control period in the intervention-first condition. The independent samples *t*-test returned no significant results between the intervention first (M = -.0003, SD=.01, 95% CIs: -.007, 0.006) and control first (M=.0018, SD = 0.14, 95% CIs: -.008, 0.011) conditions; (t (20) = -0.40, p = .690, Cohen's d = .172) suggesting the analysis could proceed as a cross-over design.

### 3.3 Period effects

Paired samples t-tests showed no evidence of a trend in sales of plant-based meals over time, with no significant differences in the mean percentage sales of plant-based meals between the first two weeks (M=.101, SD=.026, 95% CIs: 0.089, 0.112) and the second two weeks (M=.102, SD=.026, 95% CIs: 0.090, 0.113) of the study period; (t (21) = -0.41, p = .683, Cohen's d = -.088).

### 3.4 Intervention effects

**Fig. 1:**
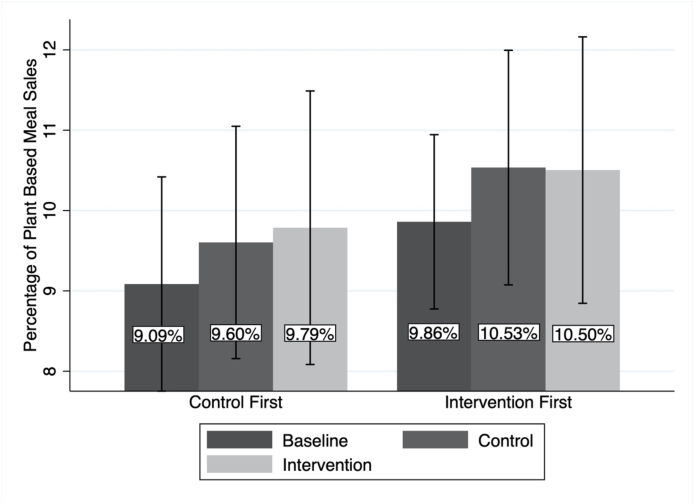
Percentage of plant-based meal sales across time (averaged for 11 restaurants in each intervention arm, with 95% CIs).

None of the independent variables (intervention condition, intervention arm and number of plant-based options available to purchase) entered into the regression model had a statistically significant effect on the percentage of plant-based meal sales, and the adjusted R^2^ value indicated that the model was not a good fit (Table 1). The results of the regression analysis did not show any evidence of an effect of the social norm messaging intervention on the percentage of plant-based meal sales (β = .0007, 95% CIs: −0.14, 0.015), controlling for the number of meal options available, and having ruled out baseline, carry over, and period effects (Fig. 1).

**Table 1:** Linear Regression Model for percentage of plant-based meal sales.

Independent Variables	β (p-value)	[CI] (95%)
Intervention Condition (Ref = Control, 1 = Intervention)	.0007(.917)	[-0.14, .015]
Intervention Arm (Ref = Control First, 1 = Intervention First)	.0075 (.219)	[-.005, .019]
No. of plant-based options (Ref = 4 options, 1 = 5 options)	.003 (.586)	[-.009, .015]
N = 66, Adj. R^2^ = −.0125 F (4,61) = 0.80

### 3.5 Intervention fidelity

Of the nine stores checked, one, three and five stores scored high, medium or low respectively on the intervention fidelity scale. Restricting the analysis only to stores with medium or high fidelity found no evidence that the plant-based sales significantly changed from control (M= .115, SD=.031, 95% CIs: 0.065, 0.165) to intervention (M=.118, SD=.033, 95% CIs: 0.065, 0.171) periods (β = .003, 95% CIs: -.027, 0.033).

## 4 Discussion

Adding a dynamic social norm message to digital screen boards placed in various locations within in-store restaurants of a major retail chain was not found to be effective in shifting customers’ choices from meat-based to plant-based meals. Controlling for the availability of meat- and plant-based dishes, and with no indication of baseline differences, carry-over, or period effects, there was no evidence of a statistically significant effect of the intervention on the percentage of plant-based meal sales. Intervention fidelity was low, but there was no evidence of an effect even when the analysis was restricted to the four stores known to have medium or high fidelity.

Previous research highlighted the importance of location, visibility, and target population of dynamic norm messages in their effectiveness (Sparkman et al., 2020). These factors were intended to be addressed in the study design by having the social message displayed in multiple prominent locations across the stores and the restaurants, animating the social message text, and consulting a focus group to refine the wording of the message. However, these considerations may not have been sufficient to ensure the effectiveness of the intervention. In the study, the dynamic social norm message was presented in a small green circle positioned on the upper right-hand corner of the images that advertised the plant-based options, which were displayed on digital screens that showed a series of images of all available meal options in a rotation. The small size and positioning of the message was decided partially due to concerns voiced by the retail partner that a bigger and more visible message could be confusing to their customers. In addition, the message could also not be displayed in the first menu screen which attracts the most customer attention because this screen was prioritized to feature the most profitable food items on the menu. Finally, though the visuals that were displayed were standardized across stores, the timing, placement and frequency of the visuals were programmed by each store manager, meaning that there could have been unpredictable inconsistencies in how and where the message was displayed that could not have been captured during the spot-check fidelity checks. The research team requested a record of the screen rotations for each site but the retail partner was unable to provide it. The non-centric location of the message, its infrequent appearance, and its comparatively small size in relation to the other visual elements on the digital screens could have decreased its salience for customers.

The customers may also have been distracted by more proximal and immediate cues in the restaurant (such as trays, cutlery, and food items on display on the buffet) rather than the images shown on the digital boards, limiting the visibility of, and exposure to, the social norm message and decreasing its ability to attract attention and incentivize customers to reflect on the information and motivate them to change their food choices. The dynamic norm message only had a loose reference group of “other customers [of the retail store]”, and the in-store restaurant-goers may not have felt enough of a social connection and identification with fellow customers visiting the store, limiting the perceived importance or desirability of performing the behavior or the need to pre-conform to the trend that was communicated. Customers could also have made food choices and eaten together with friends and family that they visited the store with, and the social norms that are imposed by these close referents in the form of their own choices and expectations could have overpowered the social norm message on display. Since we have not collected individual purchase data nor observed whether customers bought their meals alone or in the company of others, we cannot make further assumptions. Future research could consider the importance of assessing the salience of the intervention through post-intervention surveys asking customers whether they recall seeing the message and whether they remember its contents.

It is also worth noting that only 10% (range: 4–13% across 22 in-store restaurants) of meals consumed during the baseline period were plant-based, suggesting that the customer base had a strong preference for meat-based meals when visiting the in-store restaurants. The habits and cultural associations that the restaurant-goers have established with the retail chain might have been too engrained to be affected by a dynamic social norm message to which they were exposed to for only a short period of time. Habits have previously been shown to limit the success of interventions, especially since habits might prevent intentions to change from turning into actions (Verplanken & Whitmarsh, 2021). Better adherence to the intervention procedure and correct display of the message, longer and repeated exposure, increased visibility, attention-drawing design, and more strategic positioning coupled with referencing a more relevant social group, could all be considered to improve the impact of future dynamic social norm messaging interventions.

A strength of the present study is that it used a dynamic social norm message to encourage customers to replace meat-based meals with plant-based alternatives, on a much larger scale than any previous study, including all available stores of a major and well-known retail chain. The research collaboration for this study arose from informal discussions between our research staff and the retail chain's representatives. The company had an interest in promoting more environmentally sustainable food offerings, such as plant-based meals, and was open to the researchers' input into developing and evaluating behavioral interventions to support this goal. Working with a retail partner to design and implement an intervention in their locations often means a disruption to their usual business and additional responsibilities for their employees, and this collaboration was a rare and valuable opportunity. The previous studies on dynamic social norm messaging for reducing meat consumption were conducted on a university campus and its immediate surroundings, whereas here we were able to study a broad cross-section of the population. Collaborating with a retail partner enabled us to have access to 22 restaurants located across the four countries of the UK as intervention sites, increasing the reach and ecological validity of the study. The restaurants had high footfall, with several thousand meals sold weekly per store, contributing to the reliability and statistical power of the data collected from the sales. Using a randomized cross-over design allowed us to control for individual restaurant contexts and having access to baseline data on customer behavior trends prior to the intervention period allowed us to control for potential established differences across locations when measuring the efficacy of the intervention.

However, working in partnership with a commercial partner also added some restrictions to the study design. The availability of plant-based meals at the in-store restaurant was decided by the retail partner and differed between 4 and 5 options out of 13 items, constituting only 30–38% of dishes on offer. The main limitation of the study design was that not all the intervention sites were visited and checked for intervention fidelity, due to the logistic and financial constraints of visiting 22 restaurants spread across the four countries of the UK, but also following assurance from the retail chain partner that the implementation would be monitored centrally and that there would be very little scope for variability across stores on how the social norm message would be displayed on the digital boards. While no thorough fidelity checks were planned and conducted, the spot checks of the nine restaurants showed adherence was generally low, raising the possibility that exposure to the message did not occur to the extent that was intended in the original intervention design. Future research should acknowledge the complexities of collaborating with a retail partner in a real-world field experiment and should prioritize extensive fidelity checks across sites.

Communication and cooperation across the chain of command of a retail partner is important to the success of a collaboration. While the head offices of the retail partner may be very willing to work with researchers and committed to building a positive image for the company through these partnerships, managers and employees at the store level may have different priorities. To ensure that adherence to the intervention procedure remains high, positive relationships with not only the head office, but with people on the ground in the field sites, need to be established. Putting in extra effort to ensure that individual intervention sites are on board as much as the central management may help to improve intervention fidelity.

We have shown that while it is feasible to collaborate with a retail partner to implement an intervention across multiple sites, greater consideration and mitigation of potential barriers to intervention fidelity and efficacy is needed in future research. However, as implemented, there was no evidence that the intervention led to any change in plant-based meal sales across the participating restaurants, suggesting that introducing simple dynamic social norm messages may not be sufficient to alter eating behaviors in the complex food purchasing environment of the retail chain's in-store restaurants.

## Declaration of competing interest

None.

## Author contributions

KF, CS, SAJ, SH and BC designed and conducted the study. ENÇ analyzed the data and drafted the manuscript. SAJ, RP, and KF provided essential revisions to the manuscript. All authors read and approved the final manuscript.

## Funding sources

This research was funded by the 10.13039/100010269Wellcome Trust, Our Planet Our Health (Livestock, Environment and People –LEAP) award number 205212/Z/16/Z. For the purpose of Open Access, the author has applied a CC BY public copyright licence to any Author Accepted Manuscript version arising from this submission. The funders did not have a role in the study design or the collection, analysis and interpretation of the data.

## Ethics approval

As the research team was judged to be acting in an advisory role to the retail partner and not collecting any personal identifying information, University of Oxford Central University Research Ethics Committee evaluated that this study did not require ethics approval.

## Availability of data

Sales data for this research was made available on the basis of a collaboration agreement between the retail partner and the University of Oxford. Further dissemination of the study data would only be possible through expressed written permission from the retail partner.
